# Supplementary material for: Novel transgenic pigs with enhanced growth and reduced environmental impact
Source: eLife. 2018 May 22;7:e34286. doi: 10.7554/eLife.34286 (PMC5963925; doi:10.7554/eLife.34286)
Supplement: Supplementary file 1. [file elife-34286-supp1.docx]

**Supplementary File 1**. Integration site analysis of the transgenic (TG) pigs

| **Pig ID** | **Flanking region** (5'→3') | **Chromosome number** | **Integration locus** |
| --- | --- | --- | --- |
| #0707 | ATATGATTATCTTTCTAGGG**TTAA**  **GCTTTTTGAGCAGGGATTATTTTGT** | 7 | *Legumain* intron 1 |
| #0605 | ATATGATTATCTTTCTAGGG**TTAAGCTTTTTGAGCAGGGATTATTTTGT** | 7 | *Legumain* intron1 |
| #300 | ATATGATTATCTTTCTAGGG**TTAAATAAGAAAAGTTAAGATAATTAACT** | 12 | *CEP112* intron 5 |
| #301 | ATATGATTATCTTTCTAGGG**TTAAATAAGAAAAGTTAAGATAATTAACT** | 12 | *CEP112* intron 5 |
| #302 | ATATGATTATCTTTCTAGGG**TTAAATAAGAAAAGTTAAGATAATTAACT** | 12 | *CEP112* intron 5 |
| #303 | ATATGATTATCTTTCTAGGG**TTAAATAAGAAAAGTTAAGATAATTAACT** | 12 | *CEP112* intron 5 |
| #201 | ATATGATTATCTTTCTAGGG**TTAAATAAGAAAAGTTAAGATAATTAACT** | 12 | *CEP112* intron 5 |
| #202 | ATATGATTATCTTTCTAGGG**TTAAATAAGAAAAGTTAAGATAATTAACT** | 12 | *CEP112* intron 5 |
| #203 | ATATGATTATCTTTCTAGGG**TTAAATAAGAAAAGTTAAGATAATTAACT** | 12 | *CEP112* intron 5 |
| #204 | ATATGATTATCTTTCTAGGG**TTAAATAAGAAAAGTTAAGATAATTAACT** | 12 | *CEP112* intron 5 |
| #205 | ATATGATTATCTTTCTAGGG**TTAAATAAGAAAAGTTAAGATAATTAACT** | 12 | *CEP112* intron 5 |
| #501 | ATATGATTATCTTTCTAGGG**TTAAATAAGAAAAGTTAAGATAATTAACT** | 12 | *CEP112* intron 5 |
| #503 | ATATGATTATCTTTCTAGGG**TTAAATAAGAAAAGTTAAGATAATTAACT** | 12 | *CEP112* intron 5 |
| #505 | ATATGATTATCTTTCTAGGG**TTAAATAAGAAAAGTTAAGATAATTAACT** | 12 | *CEP112* intron 5 |
| #507 | ATATGATTATCTTTCTAGGG**TTAAATAAGAAAAGTTAAGATAATTAACT** | 12 | *CEP112* intron 5 |
| #703 | ATATGATTATCTTTCTAGGG**TTAAATAAGAAAAGTTAAGATAATTAACT** | 12 | *CEP112* intron 5 |
| #707 | ATATGATTATCTTTCTAGGG**TTAAATAAGAAAAGTTAAGATAATTAACT** | 12 | *CEP112* intron 5 |
| #600 | ATATGATTATCTTTCTAGGG**TTAAATAAGAAAAGTTAAGATAATTAACT** | 12 | *CEP112* intron 5 |
| #604 | ATATGATTATCTTTCTAGGG**TTAAATAAGAAAAGTTAAGATAATTAACT** | 12 | *CEP112* intron 5 |
| #708 | ATATGATTATCTTTCTAGGG**TTAA**  **AGTACTTGTGGTCCTGAGATAGTTC** | 8 | *LOC100525528* - *CXCL2* |
| #601 | ATATGATTATCTTTCTAGGG**TTAA**  **TCTAGTATACGCGTATGCCTCGAGG** | Not Detected | TG plasmid backbone |
| #603 | ATATGATTATCTTTCTAGGG**TTAA**  **TCTAGTATACGCGTATGCCTCGAGG** | Not Detected | plasmid backbone |
| #701 | ATATGATTATCTTTCTAGGG**TTAA**  **TCTAGTATACGCGTATGCCTCGAGG** | Not Detected | plasmid backbone |
| #705 | ATATGATTATCTTTCTAGGG**TTAA**  **TCTAGTATACGCGTATGCCTCGAGG** | Not Detected | plasmid backbone |
| #709 | ATATGATTATCTTTCTAGGG**TTAA**  **TCTAGTATACGCGTATGCCTCGAGG** | Not Detected | plasmid backbone |
| Control | ATATGATTATCTTTCTAGGG**TTAA**  **TCTAGTATACGCGTATGCCTCGAGG** | - | plasmid backbone |

There are 4 types of insertion patterns for the transgenes in the 25 TG founders. The TG plasmid backbone sequence is underlined. The integration loci are identified by the flanking regions from their porcine genomes, which are shown in bold.
