# Supplementary material for: Novel transgenic pigs with enhanced growth and reduced environmental impact
Source: eLife. 2018 May 22;7:e34286. doi: 10.7554/eLife.34286 (PMC5963925; doi:10.7554/eLife.34286)
Supplement: Supplementary file 2. [file elife-34286-supp2.docx]

**Supplementary file 2**. Ingredients and nutrient composition of corn-cottonseed meal-rapeseed meal-soybean meal-based (CS) and wheat-corn-soybean meal-based (WCSB) diets for examining the efficiency of nutrient utilization in transgenic grower pigs (weight range: 35–54 kg)

| **Item** | **CS diet** | **CS diet + enzymes** | **WCSB diet** | **WCSB diet + enzymes** |
| --- | --- | --- | --- | --- |
| **Ingredient, %** |  |  |  |  |
| Corn | 68.14 | 68.13 | 25.00 | 25.00 |
| Wheat |  |  | 32.46 | 32.45 |
| Wheat bran |  |  | 15.00 | 15.00 |
| Rice bran meal |  |  | 8.00 | 8.00 |
| Soybean meal (46 % crude protein) | 20.08 | 20.08 | 13.61 | 13.61 |
| Cottonseed meal | 3.00 | 3.00 |  |  |
| Rapeseed Meal | 3.00 | 3.00 |  |  |
| Soybean oil | 3.00 | 3.00 | 3.00 | 3.00 |
| Limestone | 1.32 | 1.32 | 1.25 | 1.25 |
| Sodium chloride | 0.17 | 0.17 | 0.11 | 0.11 |
| L-Lysine sulphate (L-Lys, 55%) | 0.50 | 0.50 | 0.50 | 0.50 |
| D,L-Methionine |  |  | 0.03 | 0.03 |
| L-Threonine |  |  | 0.17 | 0.17 |
| Choline chloride (60%)^1^ | 0.07 | 0.07 | 0.07 | 0.07 |
| Vitamin-trace mineral premix^2^ | 0.50 | 0.50 | 0.50 | 0.50 |
| β-glucanase, enzyme units/kg^3^ |  | 1,020 |  | 1,500 |
| Xylanase, enzyme units/kg^4^ |  | 600 |  | 1,500 |
| Phytase, enzyme units/kg^4^ |  | 1,200 |  | 1,700 |
| Titanium oxide (TiO_2_)^5^ | 0.30 | 0.30 | 0.30 | 0.30 |
| Total | 100.00 | 100.00 | 100.00 | 100.00 |
| **Analyzed or calculated nutrients (on as-fed basis)** | | | | |
| Dry matter (DM), %^6^ | 87.50 | 88.00 | 88.30 | 88.50 |
| Digestible energy (DE), kcal/kg^7^ | 3,411.76 | 3,411.76 | 3,259.8 | 3,259.8 |
| Crude protein (CP), %^6^ | 16.85 | 17.04 | 16.39 | 16.62 |
| Neutral-detergent fiber (NDF), %^6^ | 11.74 | 10.39 | 11.26 | 11.96 |
| Acid-detergent fiber (ADF), %^6^ | 4.29 | 3.9 | 6.2 | 6.44 |
| Crude fiber (%)^6^ | 2.3 | 2.1 | 3.6 | 3.7 |
| Total calcium (Ca) (%)^6^ | 0.65 | 0.7 | 5.4 | 6.2 |
| Total phosphorus (Pi) (%)^6^ | 0.37 | 0.36 | 0.57 | 0.58 |
| Available phosphorus (%)^7^ | 0.13 | 0.13 | 0.19 | 0.19 |
| Phytate phosphorus (%)^7^ | 0.26 | 0.26 | 0.36 | 0.36 |
| β-glucan (%)^8^ | 0.37 | 0.37 | 3.59 | 3.59 |
| Xylan (%)^8^ | 4.59 | 4.59 | 8.60 | 8.60 |

^1^Carried in corn cob and provided by Polestar Co., Ltd., Qingdao, China.

^2^Supplied by WENS Co., Ltd., Guangdong, China. Supplying the following micronutrients per kilogram diet (on as-fed basis): vitamin A, 6,500 IU; vitamin D_3,_ 2,000 IU; vitamin E, 42 mg; vitamin K_3,_ 2 mg; vitamin B_1_, 2 mg; vitamin B_2_, 6.4 mg; vitamin B_6_, 3 mg; vitamin B_12,_ 0.02 mg; D-biotin, 0.16 mg; D-pantothenate, 20 mg; folic acid, 1.2 mg; nicotinamide, 24; mg; Iron, 159 mg; zinc, 161 mg; copper, 142 mg; manganese, 40 mg; Iodine, 0.4 mg; selenium, 0.3 mg; and cobalt, 0.1 mg.

^3^Supplied by Shandong Longda Bio-Products Co., Ltd., Shandong, China.

^4^Supplied by Challenge Group, Beijing, China.

^5^KermeL, Tianjin, China.

^6^Analyzed value, on as-fed basis.

^7^Calculated according to the NRC (1998; 2012), Dersjant-Li et al. (2015) and Selle et al. (2003) on as-fed basis.

^8^Calculated according to the NRC (1998; 2012), on as-fed basis.
