# Supplementary material for: Novel transgenic pigs with enhanced growth and reduced environmental impact
Source: eLife. 2018 May 22;7:e34286. doi: 10.7554/eLife.34286 (PMC5963925; doi:10.7554/eLife.34286)
Supplement: Supplementary file 3. [file elife-34286-supp3.docx]

**Supplementary file 3.** Comparison of the apparent total tract nutrient digestibility values (%) between transgenic (TG) grower pigs (Line2) and their wild-type (WT) littermates fed on corn and soybean meal (CS)- and wheat, corn, or soybean meal (WCSB)- based diets with and without exogenous feed enzymes

| **Item** | **CS diet** | | | | ***P* values** |
| --- | --- | --- | --- | --- | --- |
|  | **TG** | **WT** | **WT(+)^1^** | **SEM^2^** |  |
| Average initial body weight (kg)^3^ | 34.62 | 34.33 | 33.76 | 4.36 | 0.9902 |
| Average final body weight (kg)^4^ | 41.41 | 40.25 | 39.75 | 0.63 | 0.2279 |
| Digestible energy (DE, MJ/kg)^5^ | 14.77 | 14.45 | 14.37 | 0.12 | 0.1713 |
| Neutral-detergent fiber (NDF)^4^ | 63.32 | 55.80 | 60.19 | 3.00 | 0.1317 |
| Acid-detergent fiber (ADF)^4^ | 59.36 | 51.77 | 59.95 | 3.98 | 0.3064 |
| Crude fiber (CF)^4^ | 70.14 | 63.57 | 68.08 | 2.49 | 0.1977 |
| Ash, %^4^ | 50.22^a^ | 32.69^b^ | 48.47^a^ | 3.19 | 0.0049 |
| **Item** | **WCSB diet** | | | **SEM^2^** | ***P* values** |
|  | **TG** | **WT** | **WT(+)^1^** |  |  |
| Average initial body weight (kg)^3^ | 41.80 | 40.22 | 39.75 | 4.65 | 0.9482 |
| Average final body weight (kg)^4^ | 52.75 | 54.16 | 53.91 | 0.89 | 0.5842 |
| Digestible energy (DE, MJ/kg)^5^ | 14.00 | 13.73 | 13.92 | 0.10 | 0.3446 |
| Neutral-detergent fiber (NDF)^4^ | 32.89 | 33.17 | 32.93 | 1.81 | 0.1954 |
| Acid-detergent fiber (ADF)^4^ | 34.53 | 35.49 | 32.68 | 2.49 | 0.2640 |
| Crude fiber (CF)^4^ | 55.40 | 54.22 | 55.13 | 1.60 | 0.9503 |
| Ash (%)^4^ | 50.16^a^ | 34.69^b^ | 42.69^ab^ | 0.98 | 0.0637 |

^1^WT grower pigs fed the CS and WCSB diets supplemented with an optimal dose of β-glucanase, xylanase, and phytase.

^2^Pooled standard error of the mean (n = 6).

^3^Data are expressed as the mean as analyzed by one-way ANOVA.

^4^Data are presented as least square means.

^a,b,c^Values in the same row with different superscript letters indicate statistically significant differences (ANCOVA, *P* < 0.05).

The data presented in this table can be found in Figure 3 - Source Data 2and 3
