# Supplementary material for: Novel transgenic pigs with enhanced growth and reduced environmental impact
Source: eLife. 2018 May 22;7:e34286. doi: 10.7554/eLife.34286 (PMC5963925; doi:10.7554/eLife.34286)
Supplement: Supplementary file 4. [file elife-34286-supp4.docx]

**Supplementary file 4.** Comparison of efficiency of dietary nitrogen (N), phosphorus (P), and calcium (Ca) retention (% of their dietary intake) between transgenic (TG) grower pigs (Line2) and their wild-type (WT) littermates fed on the CS and corn-soybean (CS) or wheat-corn-soybean-bran (WCSB) diets with and without exogenous feed enzymes.

| **Item** | **CS** | | | **Change**  **(%)^2^** | **SEM^3^** | ***P* values** | | |
| --- | --- | --- | --- | --- | --- | --- | --- | --- |
|  | **TG** | **WT** | **WT(+)^1^** |  |  | **TG *vs.* WT** | **TG *vs*. WT(+)** | **WT *vs*. WT(+)** |
| ***Efficiency of N retention*** | | | | | | | | |
| Fecal N output | 13.73^a^ | 18.01^b^ | 18.25^b^ | 24.00↓ | 1.37 | 0.041 | 0.023 | 0.935 |
| Urinary N output | 22.27 | 23.13 | 25.79 | - | 5.70 | 0.980 | 0.800 | 0.880 |
| Total N output | 36.00 | 41.14 | 44.04 | - | 2.14 | 0.760 | 0.460 | 0.860 |
| N retention | 64.00 | 58.86 | 55.96 | - | 2.14 | 0.760 | 0.460 | 0.860 |
| ***Efficiency of P retention*** | | | | | | | | |
| Fecal P output | 31.59^a^ | 58.25^b^ | 42.76^ab^ | 45.77↓ | 4.58 | 0.004 | 0.200 | 0.110 |
| Urinary P output | 1.10 | 0.31 | 0.31 | - | 0.41 | 0.420 | 0.510 | 0.990 |
| Total P output | 32.67^a^ | 58.56^b^ | 43.07^ab^ | 44.21↓ | 4.77 | 0.006 | 0.260 | 0.130 |
| P retention | 67.33^a^ | 41.44^b^ | 56.93^ab^ | 62.48↑ | 12.84 | 0.006 | 0.260 | 0.130 |
| ***Efficiency of Ca retention*** | | | | | | | | |
| Fecal Ca output | 32.65a | 48.32b | 35.15a | 32.4↓ | 2.85 | 0.008 | 0.847 | 0.024 |
| Urinary Ca output | 3.88^a^ | 19.36^b^ | 8.98^b^ | 79.96↓ | 1.52 | 0.000 | 0.117 | 0.001 |
| Total Ca output | 41.88^a^ | 67.68^b^ | 48.04^b^ | 38.12↓ | 4.79 | 0.006 | 0.556 | 0.044 |
| Ca retention | 58.12^a^ | 32.32^b^ | 51.96^b^ | 79.83↑ | 15.87 | 0.006 | 0.556 | 0.044 |
| **Item** | **WCSB** | | | **Change**  **(%)^2^** | **SEM^3^** | ***P* values** | | |
|  | **TG** | **WT** | **WT(+)^1^** |  |  | **TG *vs*. WT** | **TG *vs*. WT(+)** | **WT *vs*. WT(+)** |
| ***Efficiency of N retention*** | | | | | | | | |
| Fecal N output | 15.43^a^ | 20.51^b^ | 17.41^b^ | 24.77↓ | 1.31 | 0.037 | 0.055 | 0.244 |
| Urinary N output | 15.76 | 13.54 | 25.79 | - | 4.36 | 0.930 | 0.270 | 0.150 |
| Total N output | 31.19 | 34.05 | 43.20 | - | 4.17 | 0.880 | 0.140 | 0.300 |
| N retention | 68.81 | 65.95 | 56.80 | - | 4.17 | 0.880 | 0.140 | 0.300 |
| ***Efficiency of P retention*** | | | | | | | | |
| Fecal P output | 37.29^a^ | 57.17^b^ | 40.90^a^ | 34.77↓ | 1.97 | <0.0001 | 0.418 | <0.0001 |
| Urinary P output | 8.01^a^ | 0.37^b^ | 4.55^ab^ | - | 1.38 | 0.004 | 0.211 | 0.114 |
| Total P output | 45.30^a^ | 57.55^b^ | 45.46^a^ | 21.29↓ | 2.00 | 0.002 | 0.990 | 0.002 |
| P retention | 54.70^a^ | 42.45^b^ | 54.54^a^ | 28.86↑ | 2.00 | 0.002 | 0.990 | 0.002 |
| ***Efficiency of Ca retention*** | | | | | | | | |
| Fecal Ca output | 37.60^a^ | 46.42^b^ | 34.02^a^ | 19.00↓ | 1.82 | 0.010 | 0.370 | 0.001 |
| Urinary Ca output | 0.64^a^ | 6.27^b^ | 0.99^a^ | 89.79↓ | 0.57 | <0.0001 | 0.899 | <0.0001 |
| Total Ca output | 38.27^a^ | 52.69^b^ | 35.00^a^ | 27.37↓ | 1.83 | 0.000 | 0.440 | <0.0001 |
| Ca retention | 61.73^a^ | 47.31^b^ | 65.00^a^ | 30.48↑ | 1.83 | 0.000 | 0.440 | <0.0001 |

^1^WT grower pigs fed on the CS and WCSB diets supplemented with an optimal dose of β-glucanase, xylanase, and phytase.

^2^TG *vs*. WT.

^3^Pooled standard error of the mean (n = 6).

^a,b,c^Values in the same row with different superscript letters significantly differ (ANCOVA, *P* < 0.05).

The source data are presented in Figure 3-Source Data1-6.
