# Supplementary material for: Novel transgenic pigs with enhanced growth and reduced environmental impact
Source: eLife. 2018 May 22;7:e34286. doi: 10.7554/eLife.34286 (PMC5963925; doi:10.7554/eLife.34286)
Supplement: Supplementary file 5. [file elife-34286-supp5.docx]

**Supplementary file 5**. Ingredients and nutrient composition of the low nitrogen level and high proportion of phytate (78.4%) (LNHP) diet used to assess growth performances of the F1 transgenic (TG) grower gilts and the wild-type (WT) grower gilts (weight range: 30–50 kg).

| **LNHP component** | **Composition (%)** |
| --- | --- |
| Corn | 69.50 |
| Soybean meal | 12.00 |
| Wheat bran | 5.50 |
| Rapeseed meal | 3.00 |
| Cottonseed meal | 3.00 |
| Soybean oil | 2.00 |
| Limestone | 1.42 |
| Saccharose | 1.50 |
| Sodium chloride | 0.34 |
| L-Lysine sulphate (L-Lys, 55%) | 0.55 |
| DL-Methionine | 0.05 |
| L-Threonine | 0.09 |
| Choline chloride (60%)^1^ | 0.10 |
| Vitamin-trace mineral premix^2^ | 0.50 |
| Titanium oxide (TiO_2_)^3^ | 0.30 |
| Total | 100.00 |
| Digestible energy (DE), kcal/kg^4^ | 3352 |
| Crude protein (CP), %^5^ | 14.78 |
| Total calcium (Ca), %^5^ | 0.58 |
| Total phosphorus (Pi), %^5^ | 0.37 |
| Phytate phosphorus, %^5^ | 0.29 |
| Total dietary fiber (TDF), %^5^ | 9.91 |
| Neutral-detergent fiber (NDF), %^5^ | 11.4 |
| Acid-detergent fiber (ADF), %^5^ | 4.6 |
| β-Glucan, %^5^ | 0.15 |
| Xylan, %^5^ | 4.27 |

^1^Carried in corn cob and provided by Polestar Co., Ltd., Qingdao, China.

^2^Supplied by WENS Co. Ltd., Guangdong, China. Supplying the following micronutrients per kilogram of the diet (on as-fed basis): vitamin A, 6,500 IU; vitamin D, 2,000 IU; vitamin E, 40 mg; vitamin K_3_, 2 mg; vitamin B_1_, 2 mg; vitamin B_2_, 5 mg; vitamin B_2_, 6.4 mg; vitamin B_6_, 3 mg; vitamin B_12,_ 0.02 mg; D-biotin, 0.15 mg; D-pantothenate, 20 mg; folic acid, 1 mg; nicotinamide, 24 mg; iron, 134 mg; zinc, 125 mg; copper, 30 mg; manganese, 50 mg; iodine, 0.5 mg; selenium, 0.35 mg; and cobalt, 0.16 mg.

^3^KermeL, Tianjin, China.

^4^Calculated according to the NRC (1998; 2012), on an as-fed basis.

^5^Analyzed value, on an as-fed basis.
