# Supplementary material for: Novel transgenic pigs with enhanced growth and reduced environmental impact
Source: eLife. 2018 May 22;7:e34286. doi: 10.7554/eLife.34286 (PMC5963925; doi:10.7554/eLife.34286)
Supplement: Supplementary file 6. [file elife-34286-supp6.docx]

**Supplementary file 6**. Comparison of the growth performances of the F1 transgenic (TG) gilts (Line2) and the wild-type (WT) gilts fed on a low non-starch polysaccharide (NSP) diet during the growing period (weight range: 30–50 kg)

| **Item** | **WT** (n = 17) | **TG** (n = 8) | ***P* value** |
| --- | --- | --- | --- |
| ADG, g/day^1^ | 491.12 ± 81.69 | 576.38 ± 120.03^*^ | 0.05 |
| FCR, kg/kg^2^ | 2.97 ± 0.31 | 2.77 ±0.12^**^ | 0.005 |

^1^ADG, average daily gain.

^2^FCR, feed conversion ratio (i.e., average feed intake/average daily gain, kg/kg).

Asterisks indicate significant differences between the WT and the TG pigs at **P* < 0.05 (ANCOVA) or at ***P* < 0.01(ANCOVA) within the same row.

The data presented in the table can be found in Supplementary file 6-Source data1 (below)

**Supplementary file 6- Source data1**

| **Group** | **NO.** | **ADG** | **FCR** |
| --- | --- | --- | --- |
| **WT** | 828704# | 661.22 | 2.35 |
|  | 830302# | 430.06 | 3.16 |
|  | 829800# | 442.31 | 3.12 |
|  | 827600# | 599.55 | 2.45 |
|  | 832302# | 460.25 | 3.27 |
|  | 830002# | 375.06 | 3.38 |
|  | 830900# | 445.67 | 2.91 |
|  | 832502# | 431.60 | 2.97 |
|  | 830200# | 525.64 | 2.58 |
|  | 828200# | 446.31 | 3.39 |
|  | 832900# | 600.38 | 2.86 |
|  | 831202# | 515.76 | 2.91 |
|  | 850706# | 604.35 | 2.94 |
|  | 850500# | 515.38 | 2.75 |
|  | 850402# | 437.50 | 3.40 |
|  | 850400# | 439.13 | 3.07 |
|  | 850204# | 418.92 | 2.92 |
| **TG** | 850710# | 548.61 | 2.70 |
|  | 850902# | 508.52 | 2.63 |
|  | 850202# | 446.43 | 2.87 |
|  | 850504# | 529.49 | 2.70 |
|  | 850104# | 643.42 | 2.71 |
|  | 850604# | 555.88 | 2.56 |
|  | 850804# | 537.70 | 2.55 |
|  | 850700# | 840.91 | 2.85 |

Note: ADG, average daily gain; FCR, feed conversion rate(Feed/Gain).
