# Supplementary material for: Novel transgenic pigs with enhanced growth and reduced environmental impact
Source: eLife. 2018 May 22;7:e34286. doi: 10.7554/eLife.34286 (PMC5963925; doi:10.7554/eLife.34286)
Supplement: Supplementary file 7. [file elife-34286-supp7.docx]

**Supplementary file 7**. Ingredients and nutrient composition of the experimental diets that were formulated to determine the growth performance of the F2 transgenic (TG) grower-finisher pigs

| **Item** | **Grower pig** | **Finisher pig** | |
| --- | --- | --- | --- |
|  | 30–50 kg | 50–80 kg | 80–115 kg |
| Ingredient, % |  |  |  |
| Corn | 37.10 | 16.00 | 22.20 |
| Shelly barley | 18.00 | 35.90 | 22.40 |
| Wheat | 17.15 | 30.00 | 0.00 |
| Wheat bran | 0.00 | 0.00 | 17.80 |
| Standard flour | 5.00 | 0.00 | 9.50 |
| Rice bran meal | 0.00 | 0.00 | 8.80 |
| Soybean meal | 6.00 | 1.50 | 1.30 |
| Puffing whole Soybean | 12.20 | 12.00 | 13.00 |
| Soybean oil | 0.51 | 1.40 | 1.28 |
| DL-Methionine | 0.36 | 0.32 | 0.16 |
| L-Lysine (L-Lys, 55%) | 0.61 | 0.41 | 0.68 |
| L-Threonine | 0.15 | 0.09 | 0.15 |
| L-Tryptophan | 0.02 | 0.00 | 0.03 |
| Choline chloride (60%, with corn cob) | 0.10 | 0.15 | 0.20 |
| Limestone | 1.40 | 1.34 | 1.50 |
| Sodium chloride | 0.30 | 0.20 | 0.30 |
| Vitamin-trace mineral premix^1^ | 1.00 |  |  |
| Vitamin-trace mineral premix^2^ |  | 0.60 | 0.60 |
| Mold inhibitor^3^ | 0.10 | 0.10 | 0.10 |
| Total | 100.00 | 100.00 | 100.00 |
| Analyzed and calculated dietary nutrient contents (on as-fed basis) | | | |
| DE, kcal/kg^5^ | 3,400.90 | 3,386.80 | 3,101.50 |
| Crude protein (CP), %^4^ | 16.65 | 15.8 | 15.27 |
| Total calcium (Ca), %^4^ | 0.67 | 0.64 | 0.64 |
| Total phosphorus (P), %^4^ | 0.33 | 0.40 | 0.52 |
| Available phosphorus, %^5^ | 0.13 | 0.140 | 0.10 |
| Phytate phosphorus, %^5^ | 0.23 | 0.20 | 0.43 |
| Neutral-detergent fiber (NDF), %^6^ | 11.53 | 12.62 | 17.38 |
| Acid-detergent fiber (ADF), %^6^ | 4.69 | 4.57 | 6.74 |
| β-Glucan, %^6^ | 2.33 | 4.17 | 6.27 |
| Xylan, %^6^ | 5.54 | 5.67 | 8.34 |
| Soluble non-starch polysaccharides (NSP), %^6^ | 1.76 | 2.64 | 2.34 |
| Insoluble NSP, %^6^ | 10.51 | 10.31 | 14.11 |

^1^Supplied by WENS Co. Ltd., Guangdong, China. Supplying the following micronutrients per kilogram of the diet (on an as-fed basis): vitamin A, 6,500 IU; vitamin D_3,_ 2,000 IU; vitamin E, 42 mg; vitamin K_3_, 2 mg; vitamin B_1_, 2 mg; vitamin B_2_, 6.4 mg; vitamin B_6_, 3 mg; vitamin B_12_, 0.02 mg; D-biotin, 0.16 mg; D-pantothenate, 20 mg; folic acid, 1.2 mg; nicotinamide, 24 mg; iron, 159 mg; zinc, 161 mg; copper, 142 mg; manganese, 40 mg; iodine, 0.4 mg; selenium, 0.3 mg; and cobalt, 0.1 mg.

^2^Supplied by WENS Co. Ltd., Guangdong, China. Supplying the following micronutrients per kilogram of the diet (on an as-fed basis): vitamin A, 6,500 IU; vitamin D, 2,000 IU; vitamin E, 40 mg; vitamin K_3,_ 2 mg; vitamin B_1_, 2 mg; vitamin B_2_, 5 mg; vitamin B_2_, 6.4 mg; vitamin B_6_, 3 mg; vitamin B_12_, 0.02 mg; D-biotin, 0.15 mg; D-pantothenate, 20 mg; folic acid, 1 mg; nicotinamide, 24 mg; iron, 134 mg, zinc, 125 mg, copper, 30 mg, manganese, 50 mg, iodine, 0.5 mg; selenium, 0.35 mg; and cobalt, 0.16 mg.

^3^Supplied by Vega Pharma Ltd., Zhejiang, China.

^4^Analyzed value, on an as-fed basis.

^5^Calculated according to the NRC (1998; 2012), Dersjant-Li et al. (2015) and Selle et al. (2003), on an as-fed basis.

^6^Calculated according to the NRC (1998; 2012), on as-fed basis.
