# Supplementary material for: Novel transgenic pigs with enhanced growth and reduced environmental impact
Source: eLife. 2018 May 22;7:e34286. doi: 10.7554/eLife.34286 (PMC5963925; doi:10.7554/eLife.34286)
Supplement: Supplementary file 8. [file elife-34286-supp8.docx]

**Supplementary file 8**. Primers used in PCR and probes in southern blotting

| **Genomic segment** | **Strand** | **Sequences** (5'→3') | **Product size** (bp) |
| --- | --- | --- | --- |
| *BgEgXyAp* | F | CTTTCACAGTGGTCACCCAGTTTC | 1,004 |
|  | R | TCAAAGGCATAGGTATGGTAAGCG |  |
| *mPSP* *promoter* | F | GGATACTTTATTATTCTCTGACTCGGTC | 1,817 |
|  | R | CATAGTTGGTTGAAGGAATGTGTGC |  |
| *hyPBase* | F | CAGAAACATCACCTGCGACAACT | 1,785 |
|  | R | GTCCTCTTCTTCATCACGGGCTC |  |
| *Amp^r^* | F | CGGTATTTTCTCCTTACGCATCTGT | 1,897 |
|  | R | AATACGGTTATCCACAGAATCAGGG |  |
| *GAPDH* | F | CCAGCAAGAGCACGCGAGGAGGAG | 615 |
|  | R | CGGGGGTCTGGGATGGAAACTGGA |  |
| Probe | F | CGTGCAGTGCTTCAGCCGCTACCCCGACC | 610 |
|  | R | CAAGGAAGGCACGGGGGAGGGGCAAACAA |  |

Legend: F, forward; R, reverse
