# Supplementary material for: Novel transgenic pigs with enhanced growth and reduced environmental impact
Source: eLife. 2018 May 22;7:e34286. doi: 10.7554/eLife.34286 (PMC5963925; doi:10.7554/eLife.34286)
Supplement: Supplementary file 9. [file elife-34286-supp9.docx]

**Supplementary file 9**. Primers used in reverse transcription PCR, quantitative real-time PCR, and absolute quantitative real time PCR

| **Gene** | **Strand** | **Sequences** (5'→3') | **Product size** (bp) |
| --- | --- | --- | --- |
| *BgEgXyAp* | F | CTTTCACAGTGGTCACCCAGTTTC | 2270 |
|  | R | GGACTGTGGGCATCCTTTCTTAG |  |
| *bg17* | F | CTTTCACAGTGGTCACCCAGTTTC | 108 |
|  | R | TGGCGTTCTGAATCACTTTGC |  |
| *eg1314* | F | TTTACATACACCGGACCTACAGACG | 176 |
|  | R | TCAAAGGCATAGGTATGGTAAGCG |  |
| *xynB* | F | GTGACCACAAGCAACCACTTTAAC | 111 |
|  | R | GGAGGATGAGCCTGAGGACTG |  |
| *eappA* | F | AAAGCGTCGTCATCGTCTCC | 215 |
|  | R | GGACTGTGGGCATCCTTTCTTAG |  |
| *β-actin* | F | CACTGCCGCATCCTCTTCCT | 400(cDNA)  628(DNA) |
|  | R | CTCCTGCTTGCTGATCCACATC |  |

**Legend: F, forward; R, reverse**
