# Supplementary material for: Novel transgenic pigs with enhanced growth and reduced environmental impact
Source: eLife. 2018 May 22;7:e34286. doi: 10.7554/eLife.34286 (PMC5963925; doi:10.7554/eLife.34286)
Supplement: Supplementary file 10. [file elife-34286-supp10.docx]

**Supplementary file 10.** Antibody used in western blotting

| **Antibody** | **Host species** | **Epitope** | **Product size**  (kDa) |
| --- | --- | --- | --- |
| BG17A | Rabbit | QQIGRIPEVHPRLTC | 45.2 |
| XYNB | Rabbit | CSLLKQAGDVEENPG | 20.4 |
| EAPPA | Mouse | YPYDVPDYA | 45.7 |
