# Supplementary material for: Novel transgenic pigs with enhanced growth and reduced environmental impact
Source: eLife. 2018 May 22;7:e34286. doi: 10.7554/eLife.34286 (PMC5963925; doi:10.7554/eLife.34286)
Supplement: Supplementary file 11. [file elife-34286-supp11.docx]

**Supplementary file 11.** Efﬁciency of piggyBac-mediated transgenisis to produce single-copy quad-cistronic transgenic pigs

| **Donor cell** | **No. of transferred embryos** | **No. of recipients** | **No. of pregnancies (%)** | **No. of deliveries (%)** | **No. of piglets** | **No. of piglets born alive** | **No. of abnormalities** | **Cloning efficiency** | **Transgenesis efficiency** | **Transposition efficiency ^2^** |
| --- | --- | --- | --- | --- | --- | --- | --- | --- | --- | --- |
| TFF^1^ | 4,008 | 16 | 14  (87.5%) | 8  (50%) | 35 | 33 | 10 | 35/4008  (0.87%) | 25/31^2^  (80.6%) | 20/25  (80.0%) |

^1^TFF: Transgenic fetal fibroblast.

^2^ Ear samples were collected from 31 live cloned pigs at 3 days of postnatal age.

The data presented in the table can be found in Supplementary file 11-Source data1 (below)

**Supplementary file 11-Source data1**

| **Recipients** | **Breed** | **Sow parity** | **No. of transferred embryo** | **Date of delivery** | **No. of piglets** | **No. of abnormalities** | **No. of piglets born alive** | **No.of stillbirth** |
| --- | --- | --- | --- | --- | --- | --- | --- | --- |
| #10-054802 | Yorkshire | 5 | 309 | 2013/5/22 | **4** | **2** | **4** |  |
| #11-861000 | Yorkshire | 1 | 303 | no |  |  |  |  |
| #09-112802 | Yorkshire | 4 | 241 | 2013/5/24 | **6** |  | **6** |  |
| #09-114308 | Yorkshire | 6 | 231 | no |  |  |  |  |
| #12-134806 | Yorkshire | 1 | 239 | no |  |  |  |  |
| #12-850110 | Yorkshire | 1 | 251 | 2013/5/26 | **7** | **4** | **7** |  |
| #K580 | Crossbred  (Landrace x Yorkshire) | 2 | 241 | 2013/6/22 | **2** | **1** | **2** |  |
| #K512 | Crossbred  (Landrace x Yorkshire) | 2 | 266 | 2013/6/22 | **2** |  |  | **2** |
| #11-079812 | Yorkshire | 2 | 229 | no |  |  |  |  |
| #10-851208 | Yorkshire | 4 | 232 | 2013/7/7 | **5** |  | **5** |  |
| #11-062804 | Yorkshire | 2 | 229 | no |  |  |  |  |
| #10-156802 | Yorkshire | 5 | 247 | no |  |  |  |  |
| #12-003502 | Yorkshire | 0 | 266 | no |  |  |  |  |
| #10-113706 | Yorkshire | 2 | 210 | 2013/2/2 | **5** | **1** | **5** |  |
| #11-808504 | Yorkshire | 2 | 217 | 2013/2/2 | **4** | **2** | **4** |  |
| #11-808302 | Yorkshire | 2 | 297 | no |  |  |  |  |
|  | | | | **total** | **35** | **10** | **33** | **2** |
